# Suicide gene therapy for the treatment of high-grade glioma: past lessons, present trends, and future prospects

**DOI:** 10.1093/noajnl/vdaa013

**Published:** 2020-02-06

**Authors:** Jubayer A Hossain, Antonio Marchini, Boris Fehse, Rolf Bjerkvig, Hrvoje Miletic

**Affiliations:** 1 Department of Biomedicine, University of Bergen, Bergen, Norway; 2 Haukeland University Hospital, Bergen, Norway; 3 Department of Oncology, Luxembourg Institute of Health, Strassen, Luxembourg; 4 German Cancer Research Center (DKFZ), Heidelberg, Germany; 5 Department of Stem Cell Transplantation, University Medical Center Hamburg-Eppendorf, Hamburg, Germany

**Keywords:** glioma, immunotherapy, stem cells, suicide gene therapy, viral vectors

## Abstract

Suicide gene therapy has represented an experimental cancer treatment modality for nearly 40 years. Among the various cancers experimentally treated by suicide gene therapy, high-grade gliomas have been the most prominent both in preclinical and clinical settings. Failure of a number of promising suicide gene therapy strategies in the clinic pointed toward a bleak future of this approach for the treatment of high-grade gliomas. Nevertheless, the development of new vectors and suicide genes, better prodrugs, more efficient delivery systems, and new combinatorial strategies represent active research areas that may eventually lead to better efficacy of suicide gene therapy. These trends are evident by the current increasing focus on suicide gene therapy for high-grade glioma treatment both in the laboratory and in the clinic. In this review, we give an overview of different suicide gene therapy approaches for glioma treatment and discuss clinical trials, delivery issues, and immune responses.

High-grade gliomas (HGGs), collectively known as WHO grade III and IV primary brain tumors, belong to the most deadly and incurable group of cancers.^1^ Lack of specificity of chemotherapeutic drugs, delivery issues to the central nervous system, and development of therapy resistance pose challenges in glioma management. The urgent need for novel and more efficient treatment strategies has produced various novel molecularly targeted therapeutic options that have been tested in clinical trials alongside the traditional chemotherapy/radiation approaches.^2^ However, the outcomes have mostly fallen short of expectations.^2,3^ Another experimental treatment modality that has persistently been tested in HGG patients is suicide gene therapy (SGT) or also known as gene-directed enzyme prodrug therapy. The initial successful proof of concept in animal models^4^ and small-scale clinical trials^5^ could not be replicated in a large-scale trial^6^ generating a negative wave against further development of SGT. However, continued research has promoted further improvements of this strategy resulting in a more tailored therapeutic avenue for HGG. SGT is a multi-componential approach (Figure 1) and thus offers unique possibilities of tailoring the therapy further by improving the individual components according to the new mechanistic insights into glioma biology, novel vector developments, and also the development of new delivery techniques such as convection-enhanced delivery (CED). Therefore, these new developments have been molding SGT to become potentially more effective for HGG treatment. As a result, several improved SGT systems are currently being tested in the laboratory and some have entered clinical trials. In this review, we discuss past and present developments in the SGT field and also critically review the clinical translation, delivery issues, and immune responses.

**Fig. 1:**
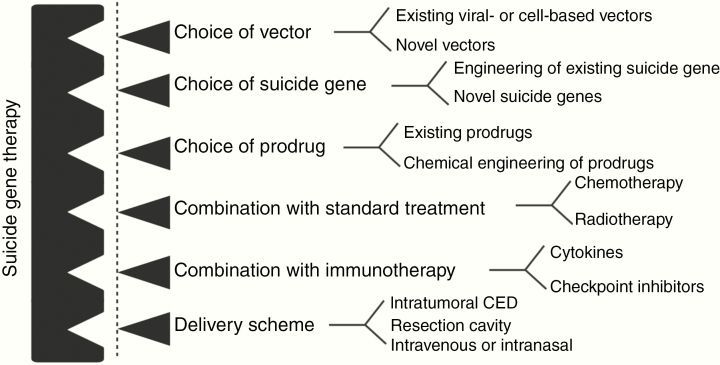
SGT consists of different modules where each single module can be subjected to improvement to enhance therapeutic efficacy. CED, convection-enhanced delivery.

## The Concept of SGT

**Fig. 2:**
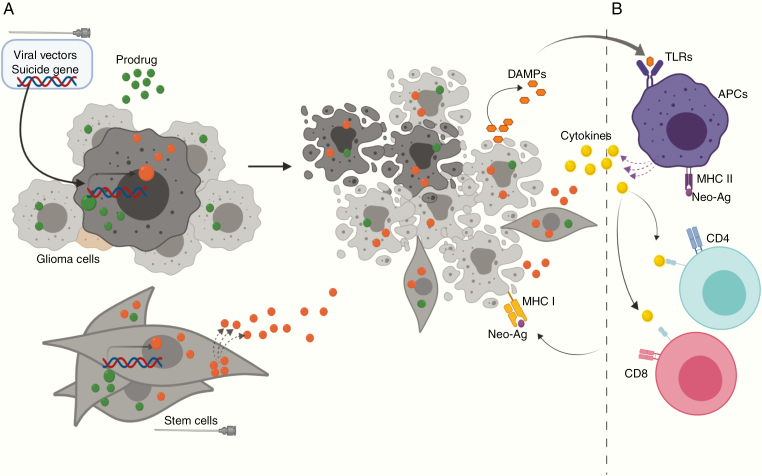
The basic mechanism of SGT. (A) The suicide gene is delivered into glioma cells by viral vectors that convert the nontoxic prodrug (green) into a toxic metabolite (orange) that causes tumor cell death. Note that the toxic metabolite (or intermediate byproducts) can travel from the transduced tumor cells (dark) to the untransduced tumor cells (light) by either gap junctions or diffusion, finally leading to the death of both transduced and untransduced cells. This phenomenon is known as the bystander effect (BE). The precise mechanism of BE is dependent on the nature of the toxic drug. Suicide gene-modified stem cells kill the tumor cells via BE only. (B) Recent studies suggest that SGT can cause a release of damage-associated molecular pattern (DAMP) molecules and/or can induce a display of neo-antigens (neo-Ags) leading to immunogenic cell death. Both myeloid antigen-presenting cells (APCs) and lymphocytes are instrumental in the resulting antitumor immune response.

SGT represents the most common form of gene therapy used to treat HGGs in both preclinical and clinical settings. From a theoretical perspective, SGT is a two-step treatment modality for solid tumors (Figure 2A): the first step is the transduction of cancer cells by a vector encoding an enzyme (suicide gene) capable of catalyzing a prodrug into a toxic metabolite. The second step involves administration of the corresponding prodrug that upon catalysis by the prodrug-converting enzyme induces cell death. Ideally the prodrug should exhibit (1) features of an ideal substrate for the enzyme, (2) activation of cell death after catalysis with minimal or no off-target toxicity, (3) capability of crossing the blood–brain barrier efficiently, and (4) induction of the so-called bystander effect (BE). Since 100% transduction of all tumor cells is virtually impossible, the BE is an important feature of SGT. It facilitates collateral killing of non-transduced (“bystander”) cells caused by the transfer of intermediate or final metabolites of the prodrug. Thus, it is sufficient to transduce a certain fraction of the tumor to potentially achieve complete eradication of all malignant cells. Growing evidence indicates that the process of cell death induced by certain SGTs is immunogenic, which means that it can alert and stimulate an antitumor response adding to the treatment effect of SGT (Figure 2B).

## Development of Suicide Genes and Prodrugs

**Fig. 3:**
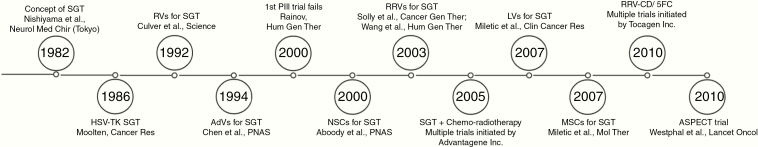
Timeline of major developments in SGT for HGG treatment.

The possibility of using microbial enzymes and an antimicrobial compound to introduce selective cytotoxicity in mammalian cells was first reported in the 1980s demonstrating that transfer of genetic material of the Herpes simplex virus 1 (HSV), rendered the cells more sensitive to acyclovir (ACV)^7^ (Figure 3). Nishiyama et al.^8,9^ adopted this concept for cancer treatment by showing that delivery of cytosine deaminase (CD) from *Escherichia coli* followed by 5-fluorocytosine (5FC) administration leads to a significant reduction of tumor burden in a syngeneic EA285 rat glioma model. Moolten et al.^10,11^ contemporarily introduced SGT based on thymidine kinase (TK) from HSV for cancer treatment (Figure 3). *CD* and *HSV-TKHSV-TK* are the two most widely used suicide genes for cancer treatment including HGGs. However, a wide variety of other SGTs have been *HSV-TK* established (Table 1).

**Table 1.** List of Most Prominent Suicide Gene Therapy Systems Used for HGG Treatment

Suicide Gene	Origin of Suicide Gene	Prodrug/Drug	PMID
HSV-TK	Viral	GCV/GCV-TP	19617915
VZV-TK	Viral	GCV/GCV-TP	9231072
Tomato-TK	Viral	AZT/AZT-TP	20154339
EHV4-TK	Viral	GCV/GCV-TP	12489026
Cytosine deaminase	Bacterial and yeast	5-FC/5-FU	23969884
Purine nucleoside phosphorylase (PNP)	Bacterial	MeP-dR/MEP	15374975
Nitroreductase	Bacterial	CB1954/AHNB	27840931
Guanine phosphorybosyl transferase	Bacterial	6TX/6GMP	9414253
Carboxylesterases (CE)	Mammalian	IRT/SN-38	24167321
Cytochrome P450	Mammalian, rodent	CPA/PM	9354446

While some suicide genes are compatible with several prodrugs, only one representative prodrug along with the corresponding toxic drug is mentioned here.

GCV-TP, GCV triphosphate; MeP-dR, 9-β-d-[2–deoxyribofuranosyl]-6-methylpurine; MEP, 6-methylpurine; CB1954, 5-aziridinyl-2,4-dinitrobenzamide; AHNB, 5-(aziridinyl)-4-hidroxylamine-2-nitrobenzamide; IRT, irinotecan; 6TX, 6-thioxanthine; 6GMP, 6-thioguanine monophosphate; CPAC, cyclophosphamide; PM, phosphoramide mustard.

### CD-Based SGT

CD, an enzyme found in bacteria and lower eukaryotes (eg, yeast), is involved in microbial pyrimidine metabolism and deaminates 5FC (and other analogs, namely, 6-azacytosine, isocytosine) into 5-fluorouracil (5FU). 5FU is a pyrimidine analog that directly inhibits nucleic acid synthesis due to misincorporation instead of uracil or thymine. 5FU can also be catalyzed by cellular enzymes into fluorodeoxyuridine monophosphate that can interfere with DNA metabolism by binding thymidylate synthase.^12^ Therefore, 5FU itself has been used as a potent anticancer chemotherapeutic agent for many years, but off-target toxicity has limited its direct applicability.^8,9,13^ Two different CD proteins have been adopted for developing the SGT system with CD/5FC: bacterial CD (bCD; source: *E. coli*) and yeast CD (yCD; source: *Saccharomyces cerevisiae*). Although bCD and yCD bear little homology, they catalyze cytosine and 5FC in a similar fashion, albeit with different efficiency. yCD has a significantly lower *K*_m_ and a higher *V*_max_ for 5FC than bCD^13^; however, yCD exhibits thermal instability. The enzyme works optimally at around 26°C and loses activity as temperature rises.^8,13^ This shortcoming has been mended by rational protein engineering that involves alteration of 3 amino acids in the *yCD* gene.^14^ Currently, both bCD and the recombinant yCD are being used for HGG treatment.^15,16^

### HSV-TK-Based SGT

HSV encodes a *TK* gene that is evolutionarily and functionally different than the human thymidine kinases (hTKs).^17^ Compared to hTKs, HSV-TK more efficiently catalyzes various prodrugs (synthetic nucleoside analogs) producing mono-phosphorylated nucleoside analogs that are further phosphorylated by cellular kinases.^17^ The resulting triphosphorylated analogs are incorporated into DNA strands during replication and cause strand abrogation leading to cell death of actively proliferating cells. Importantly, the analogs (ie, prodrugs) are not efficiently recognized by the hTKs preventing toxicity for normal cells. As a result, HSV-TK acts as a suicide gene upon prodrug exposure without any major interference of the hTKs. Various purine and pyrimidine analogs are compatible with HSV-TK SGT such as ganciclovir (GCV), ACV, and brivudin (BVDU).^18,19^ BVDU is an efficient substrate of HSV-TK and a potent inducer of cell death,^20^ but exhibits poor BE.^21^ GCV is a better substrate for HSV-TK compared with ACV and exhibits a greater BE compared to either ACV or BVDU.^21,22^ Valganciclovir (valGCV), an oral analog of GCV, has recently been shown to be suitable for long-term treatment in a GBM xenograft model.^23^

The wild-type HSV-TK suffers from a few shortcomings: higher affinity toward its natural substrate endogenous thymidine (dT) compared to GCV^24^ and presence of cryptic sites leading to anomalous transcription^25^ or splicing.^26^ Such limitations can be overcome by optimizing sequences of the *HSV-TK* gene,^27^ and a novel mutant with superior functionality has been developed to be used for treatment of experimental HGG.^17,23,28,29^

## Vector Systems for SGT

Soon after the emergence of SGT principles,^9,10^ γ-retroviral vectors (RVs) and later adenoviral vectors (AdVs) were employed to deliver suicide genes into tumors.^4,30^ Although these vectors were effective in glioma animal models and to some extent in early-phase clinical trials, results from a larger phase III trials were disappointing.^6,31^ Treatment failure was mostly attributed to various shortcomings of the viral vectors indicating that more efficient vector systems need to be developed to harness the power of SGT for cancer treatment. Thus, per today a wide variety of vectors derived from different viral backbones are used for SGT (Table 2). Apart from viral vectors, stem-cell-based vectors have been developed for SGT.

**Table 2.**
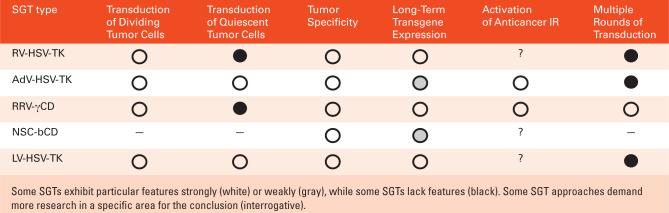
Key Features of an Efficient SGT System for HGG Treatment

### AdV-Mediated SGT

The failure of RV-mediated SGT in a phase III clinical trial^6^ was attributed to low transduction efficiency which gave an impetus to the development of viral vectors with better transduction capability. Since AdVs can transduce non-dividing cells, it was anticipated that the problem of suboptimal transduction of RVs would be solved by using AdVs. Indeed, non-replicating AdVs have been demonstrated to deliver a transgene more efficiently than RVs in human gliomas.^32^ In line with this, AdV-mediated HSV-TK/GCV (AdV-TK/GCV) therapy demonstrated a significant therapeutic benefit in experimental glioma models by several independent investigators^18,33,34^ and also in small-scale HGG clinical trials.^35–37^ No serious adverse effects were observed, which is an important finding since AdVs had caused death of a patient in a trial for ornithine transcarbamylase gene therapy.^38^ Thus, a phase III clinical trial (known as ASPECT) was performed to evaluate the potential therapeutic benefit of AdV-TK/GCV therapy as an adjuvant treatment arm to standard of care (resection, radiotherapy, and temozolomide [TMZ] administration).^31^ While ASPECT showed significant improvement in “time-to-re-intervention” compared to standard care, unfortunately the study failed to show a significant effect on overall survival. Although there was a stronger treatment effect in patients with non-methylated MGMT (*O*-6-methylguanine-DNA methyltransferase; a DNA repair protein and a major prognostic factor for TMZ treatment), the results did not satisfy the European Medicines Agency (EMA) and thus the marketing request was denied. Several theoretical reasons could be attributed to the failure of the ASPECT trial. Firstly, it is possible that the interpretation of the therapeutic outcome of the SGT was compromised by the lack of universal standard care protocols (namely, differential TMZ administration) across all institutions.^31,39^ Secondly, the therapy itself might not have been effective enough. Despite improved transduction capability, AdVs suffer from a major drawback by the lack of long-term expression of transgenes. Unlike RVs, AdVs do not integrate into the host genome and thus transgenes are only expressed transiently for up to a few weeks.^38,40^ In this context, we have recently shown that short-term SGT can result in treatment-escape and that long-term suicide gene activity can improve treatment efficacy.^23^ While the problem of short-term gene expression can be somewhat mitigated in the CNS where the transgene expression may be detected for at least up to 3 months,^18^ the episomal maintenance of AdVs still causes the transgene expression to be reduced following cell divisions that would likely lead to compromised efficacy during tumor expansion.

Because of its excellent safety profile, high transduction efficiency in both dividing and non-dividing cells, standardized manufacturing process of clinical batches, and potential to immunogenic stimulation (discussed below), AdV-mediated SGT is still pursued in both laboratories and the clinic with different combinatorial HGG treatment strategies. In this context, concomitant radiotherapy with AdV-TK/ACV has recently been tested in clinical trials showing notable improvement in survival outcomes.^19,41,42^ Other combination strategies such as concomitant TMZ or Nivolumab (checkpoint inhibitor) are also being pursued in different clinical trials at this point (Table 3). Another combinatorial paradigm involves dual AdV therapy where the second vector delivers the immunostimulatory gene Flt3L that increases antitumor immune responses (Table 3).^43,44^

**Table 3.** Clinical Trials Involving SGT for HGG Treatment

Trial No.	Start– Completion^a^	Phase	Patients	Vector Used	Suicide Gene/ Prodrug	Combination	Result	Citation
NCT00751270	2005–2011	Ib	Newly diagnosed HGG	AdV	HSV1-TK/ valACV	RT+TMZ^b^	Safety assessed	^20^
NCT00589875	2007–2015	Iia	Newly diagnosed HGG	AdV	HSV1-TK/ valACV	RT+TMZ^b^	Safety assessed	^45^
NCT00870181	2008–2012	II	Recurrent HGG	AdV	HSV1-TK/ GCV	N/A	Improved survival	^42^
NCT01172964	2010–2015	I	Recurrent HGG	NSC(HB1. F3.CD)	bCD/5FC		Safety assessed	^46^
NCT00634231	2010–2015	I	Newly diagnosed HGG (pediatric)	AdV	HSV1-TK/ valACV	RT+TMZ^b^	Safety assessed/ ongoing	^47^
NCT01156584	2010–2016	I	Recurrent HGG	RRV	yCD/5FC	N/A	—	—
NCT01470794	2012–2016	I	Recurrent HGG (undergoing surgery)	RRV	yCD/5FC	N/A	Safety + encouraging efficacy	^48,49^
NCT01985256	2014–2016	I	Recurrent HGG (undergoing surgery)	RRV^c^	yCD/5FC	N/A	—	—
NCT02015819	2014–2019	I	Recurrent HGG	NSC(HB1. F3.CD)	bCD/5FC+ Leucovorin	N/A	Ongoing	
NCT01811992	2014–2020	I	Newly diagnosed HGG	AdV	HSV1-TK/ valACV	AdV-Flt3L	Ongoing	—
NCT02414165	2015–2019	II-III	Recurrent HGG	RRV	yCD/5FC	—	—	^50^
NCT02192359	2016–2020	I	Recurrent HGG	HB1. F3.CD21. hCE1m6	hCE1m6/ irinotecan	—	Ongoing	—
NCT03596086	2017–2023	I-II	Recurrent HGG	AdV	HSV1-TK/ valACV	RT+TMZ	Ongoing	—
NCT03603405	2018–2023	I-II	Newly diagnosed HGG	AdV	HSV1-TK/ valACV	RT+TMZ	Ongoing	—
NCT03576612	2018–2021	PI	Newly diagnosed HGG	AdV	HSV1-TK/ valACV	RT+ Nivolumab+TMZ^b^	Ongoing	—
NCT02598011	2016–2022	I	Newly diagnosed HGG	RRV	yCD/5FC	RT+TMZ	Planned	—

Only the trials carried on/undertaken/planned since 2010 are mentioned here. See review from Kaufmann et al.^36^ where some of the trials before 2010 are discussed.

^a^Primary completion.

^b^TMZ allowed after prodrug administration.

^c^Intravenous administration.

### Replicating Retroviral Vector-Mediated SGT

The major drawbacks of RVs and AdVs, namely, low transduction rate and episomal nature may be circumvented by using replicating retroviral vectors (RRVs). RRVs of non-primate origin have been reported to efficiently transduce glioma cells and thus refocused the translational attention of SGT involving γ-RVs.^51^ Since RRVs exhibit a non-lytic life cycle, the therapy is mostly dependent on the suicide gene activity which could be achieved by using CD or viral TK.^45,51^ While RRVs in general transduce only dividing cells, the high-transduction ability is conferred by the replicative nature of the vectors. Most importantly, RRV replication is restricted to glioma cells in vivo due to the post-mitotic state of most normal cells within the CNS.^51^ By using a recombinant *yCD* gene,^47^ several clinical trials funded by Tocagen, Inc., were performed demonstrating safety as well as encouraging results compared to external lomustine-treated cohorts.^51,52^ These positive indications resulted in the designation of breakthrough and PRIME status for HGG treatment by FDA and EMA, respectively. The clinical studies took advantage of next-generation sequencing technologies revealing some interesting aspects of the therapy in relation to associated prognostic factors. For example, an important discovery was made by identifying a transcriptomic signature, termed survival-related neuronal subtype (SRNS), that is associated with Toca 511/5FC-mediated survival.^51^ Interestingly, the SRNS signature shows functional similarities with the TCGA neural subtype and thus patients who exhibited SRNS (and a TCGA neural subtype) benefitted most from Toca 511/5FC. Aside from SRNS, some other prognostic factors were also identified including a tentative identification of *Isocitrate dehydrogenase 1 (IDH1)* mutation as a positive prognostic factor.^51,52^ Furthermore, the study revealed that the activity of CD/5FC, similar to HSV-TK/GCV, is independent of patient MGMT status. The preliminary success warranted a phase III clinical trial (NCT02598011) which has been performed in 403 HGG patients. Recently Tocagen, Inc., made a press release announcing that the phase III trial unfortunately failed to meet the study endpoints.^53^ While detailed results are currently unavailable, the failure once again reveals the tremendous challenge of treating HGGs. Further improvement related to this treatment strategy may most likely depend on combinatorial approaches with other treatment modalities.

### Lentiviral Vector-Mediated HSV-TK/GCV Therapy

Lentiviral vectors (LVs), developed as a spin-off from HIV research, serve as one of the most popular vector systems in gene therapy. In contrast to the aforementioned vectors, LVs offer 2 unique features that are very important for SGT toward HGGs: transduction capability in quiescent cells and long-term gene expression. Similar to all retroviruses, lentiviruses integrate the provirus into host-cell genomes. However, unlike γ-retroviruses, lentiviruses are equipped with active nuclear transport machinery leading to genome integration independent of mitosis. LVs, availing this mechanism, can transduce quiescent glioma cells much more efficiently than RVs, as shown in biopsy-based glioma spheroids,^48^ where a significant fraction of non-dividing tumor cells is present. These resting glioma cells could not be targeted by RVs neither in vitro nor in vivo. LVs are most frequently pseudotyped with the glycoprotein (GP) of vesicular stomatitis virus (VSV-G). However, GPs from other viruses can also be used. We have shown that LVs pseudotyped with the GPs of the lymphocytic choriomeningitis virus transduce glioma cells more specifically compared to VSV-G pseudotyped vectors which have a strong tropism for neurons.^54^ To investigate therapeutic efficacy, LV-based HSV-TK/GCV treatment was tested in a patient-derived GBM xenograft model and subsequently complete, albeit temporary, tumor remission after GCV administration was observed.^48^ In this study, a fraction of tumor cells from recurrent tumors still expressed the suicide gene indicating that short-term prodrug administration (2–3 weeks), which is currently standard in clinical trials, is not sufficient to achieve the maximum treatment effect. This hypothesis was confirmed in a follow-up study showing that long-term administration of valGCV, a prodrug tailored for oral application, increased the treatment effect compared to short-term GCV treatment.^23^ Due to unrestricted transducing potential, LVs, in particular those pseudotyped with VSV-G, can also transduce normal post-mitotic brain cells, however, without toxicity even when using HSV-TK/GCV.^55^ On the contrary, normal brain cells expressing HSV-TK contribute in eliminating glioma cells through BE.^49^ LV-based SGT has not yet been tested in clinical trials; however, the various auspicious features observed in the most clinically relevant GBM models strongly warrant clinical investigation.

### Cell-Based SGT

Apart from viral vectors, different types of cells are also used as vectors for SGT. Stem cells such as neural stem cells (NSCs) and mesenchymal stem cells (MSCs) are the major sources for this strategy. Recently, olfactory ensheathing cells have also been shown to be an efficient vector for SGT.^50^ In general, these cells show an intrinsic migratory capacity and exhibit exceptional tropism toward pathological conditions including neoplastic lesions in the CNS.^50,56^ The tumor-tropic property of these cells has its origin in the bona fide regenerative and reparative roles in cellular homeostasis which also relates to the sensing of various pro-tumorigenic signals such as angiogenesis, hypoxia, inflammatory signals, etc..^50,57–60^ Furthermore, these cells survive in vivo engraftment (even in an allogeneic situation) for a certain time period due to low or undetectable MHC expression^56^ and do not form neoplastic lesions indicating a high safety profile.^56^ Cell-mediated tumor-killing activity solely depends on the BE and thus an SGT system with high bystander efficiency is a prerequisite.^57,61^ NSCs were the first type of cells used as a vector for SGT. The delivery potential of NSCs for SGT of glioma was first reported by using an immortalized murine NSC line which was retrovirally transduced with bCD. The engrafted NSCs migrated in a glioma-specific manner, both ipsilaterally and contralaterally, and were able to kill tumor cells upon 5FC administration.^15^ An important issue for clinical translation of NSC-mediated SGT (or any cell-based system for that matter) is to choose between an autologous and allogeneic source. Ideally an autologous source of NSC will be preferential based on immune escape. Although NSCs normally show low levels of MHC expression, there exists an immunogenic potential^62^ that would eventually promote the clearing of allogeneic NSCs within week(s).^56^ This issue can be partially circumvented by using immunosuppressive drugs; however, this may thwart anticancer immune responses and interfere with the overall treatment efficacy. Furthermore, the use of autologous NSCs is associated with several logistic shortcomings such as lack of adequate source as well as long-term culture for expansion to large cell numbers for clinical application. Cellular reprogramming technologies such as induced pluripotent stem cells are currently being pursued to obtain sufficient cell numbers of autologous NSCs.^63,64^ In contrast, the use of allogeneic NSCs offers several advantages over autologous NSCs in terms of time, cost, scalability, and standardization procedures.^57^ To date, two different NSC-mediated SGTs have been pursued in clinical studies and both are based on an immortalized allogeneic human NSC line known as HB1.F3.^65,66^ The first trial involved treatment with a bCD-modified HB1.F3 cell line in order to evaluate initial safety and feasibility.^67^ The NSCs were observed to migrate to distant tumor sites in the HGG patients and initial safety was demonstrated. As a result, a phase I trial with 18 patients has been started which will be completed soon (Table 3). The second trial involves the HB1.F3 line expressing the suicide gene human carboxylesterase (hCE1m6) (Table 3).

MSCs^68^ possess an intrinsic migratory capacity toward pathological lesions similar to NSCs and in this regard no substantial differences have been found between these two cell types.^69^ In addition, MSCs can be derived from various tissues and organs such as bone marrow, adipose tissue, umbilical cord blood, and placenta^68^ offering better accessibility for procurement compared to NSCs. Furthermore, MSCs can be easily expanded to high cell numbers for clinical application. Thus, the accessibility and also scalability of MSCs can provide advantages over NSCs for treatment application. The first SGT approach using MSCs for HGG treatment was reported by Miletic et al.^70^ in an orthotopic, syngeneic rat glioma model. The study showed a substantial treatment effect of intratumorally injected MSCs termed bone-marrow-derived tumor-infiltrating cells expressing HSV-TK following prodrug treatment. The direct tumor cell killing by MSCs was mediated through BE, while an immune response with infiltration of T cells and NK cells was detected in the treated tumors, which may have contributed to the treatment effect.^70^ Since then a number of preclinical studies have been published.^46,71^ However, no clinical trial has been performed yet for HGG treatment.

## Delivery of SGT Vectors

Delivery of gene therapeutic products into brain tumors is an important issue that is critically discussed in the field, however still lacks optimal solutions. Systemic delivery of viral vectors is feasible^72^ but challenging due to potential off-target transduction in non-CNS tissues and/or insufficient bioavailability in the CNS. Intranasal delivery of cell-based vectors has also been pursued successfully in preclinical models.^50,73^ However, intracranial injection has been the most popular mode of administration and for the majority of SGT trials so far vectors have been injected directly into the resection cavity after surgery using multiple injections.^6,31,51^ This method of application is suboptimal as the tissue around the resection cavity is very heterogeneous containing either diffusely infiltrating tumor cells or reactive brain tissue. Thus, there is no control of how much tumor tissue is reached with this method, which might also partly explain the failure of clinical trials. There is a huge interpatient variation concerning injection efficacy and the amount of target tissue reached, which makes interpretation of clinical trial data extremely difficult. CED is a sophisticated delivery method into solid tissue that has been developed in particular for the brain and also brain tumors.^74^ CED is applied through stereotactically placed catheters that are connected to a micropump maintaining a continuous low-pressure flow into the tissue. This method has substantially increased the amount of tissue that can be targeted and thus is frequently used to inject vectors or drugs into the brain.^74^ Regarding the treatment of brain tumors, this method is optimized for application into solid tumor tissue, however not into a resection cavity after surgery. The problem that emerges from here is that primary tumors are usually treated by neurosurgery that is the standard of care. Thus, at this point, the only choice in order to implement CED into future clinical trials is to inject the vectors either into recurrent tumors or inoperable primary tumors.

## Interaction of Glioma SGT With the Immune System and Combination With Immunotherapy

In general, potential antitumor immune responses mediated by SGT can originate from its different modules such as the type of vector, the cell death mechanism following suicide activity, the type of prodrug, the immune microenvironment of the tumor, and any additional treatment regimen. Different types of vector systems can have a variable impact on the immune system. While RRVs, LVs, and NSCs are not highly immunogenic in nature,^16,55,57,58,75^ AdVs are capable of eliciting an acute immune response including secretion of several proinflammatory cytokines.^18^ However, vector-induced immune responses can be a double-edged sword with either limiting transgene delivery and thereby impeding treatment efficacy or in contrast potentially breaking the immune tolerance of the glioma microenvironment^76^ and thereby enhancing the treatment effect of SGT.

The mechanism of HSV-TK/prodrug-mediated cell death can be variable involving necrotic and immunogenic cell death (ICD) in melanoma cells, but apoptotic and non-ICD in colorectal cancer cells.^77^ Glioma cells also undergo HSV-TK/prodrug-mediated cell death via apoptosis.^78,79^ The immunogenicity of apoptosis in general is controversial and not yet fully explored, in particular not in the context of SGT. Considering the plastic nature of leukocytes and their dichotomous role in antitumor IR,^80,81^ more studies are warranted to unravel the exact nature of HSV-TK/GCV-mediated cell death from an immunogenic point of view.

AdV-HSV-TK SGT has been shown to cause infiltration of various immune cells including macrophages and T cells in both rodent models^18,30^ and clinical settings.^19,82^ Still, in glioma, the antitumor IR elicited by AdV-HSV-TK/GCV therapy is often not strong enough without additional immunostimulatory strategies.^43^ When boosted by the co-expression of Flt3L or treatment with checkpoint inhibitors such as anti-PD1 antibodies, AdV-HSV-TK/GCV elicits a more robust antitumor IR.^43,44,83–85^ The co-expression of Flt3L has been shown to recruit dendritic cells to the tumor microenvironment and thereby increase antigen presentation and subsequently T-cell infiltration and activation in glioma animal models.^43,44,83^ This strategy is currently being investigated in phase I clinical trial (Table 3).

Toca 511 has been shown to activate an antitumor immune response in murine glioma cells where CD4+ T cells seemed to be crucial.^16,86^ While the precise nature of cell death due to yCD/5FC is not known, Toca 511 generated a strong antitumor immune response with an immunological memory that rejected a subsequent xenograft of the same tumor.^16^ This immune response was shown to be associated with reduced myeloid-derived suppressor cells and regulatory T cells in the tumor microenvironment.^16,86^ By conducting the adoptive transfer of splenocytes from cured mice, Mitchell et al.^86^ demonstrated that a T-cell-mediated antitumor immunity can be transferred to the host.

To conclude, a number of studies indicate that SGT can elicit an antitumor immune response (Figure 2B). While some of the underlying mechanisms of this causality have been identified, there remain several open questions highlighting the need for more fundamental research. Disappointing results of the clinical trials with AdV-HSV-TK/GCV^31^ and RRV-yCD/5FC^53^ further highlight the gravity of these issues.

## Future Perspectives

Over the last decade(s), new developments in SGT have emerged, in particular new vectors and suicide genes with improved affinity to prodrugs (Figure 3). There are, however, some important issues that should be considered for further improvement of SGTs, which is urgently needed as larger phase III clinical trials have failed so far. For instance, the preclinical model systems should be revisited. The serum-culture-based patient-derived-xenografts that have been used so far to develop various SGTs, namely, AdVs and RRVs, neither share the genetics nor the invasive features of human gliomas in patients.^87^ Another crucial difference is the proliferative index. U87, one of the most popular serum-culture-based glioma cell lines, shows an extreme in vivo proliferative index of up to 80%.^88^ In contrast, the median proliferative index of human glioblastoma in patients is about 27%.^89^ Thus, quiescent glioma cells, which often have been associated with a stem cell and resistant phenotype, pose an enormous challenge to γ-RV-based SGTs which only transduce actively dividing cells. The RRVs have not been tested either in patient-derived primary spheroid models^87,90^ or glioma stem cell lines (GSCs) which contain such quiescent cells and are considered as the new standard for preclinical studies in gliomas.^87,91^ Another important issue is the delivery of the vectors as discussed above. CED could overcome the poor distribution of viral vectors; however, the presence of a solid tumor mass instead of a resection cavity is clearly preferred. Preclinical studies should be performed to test this hypothesis.

**Fig. 4:**
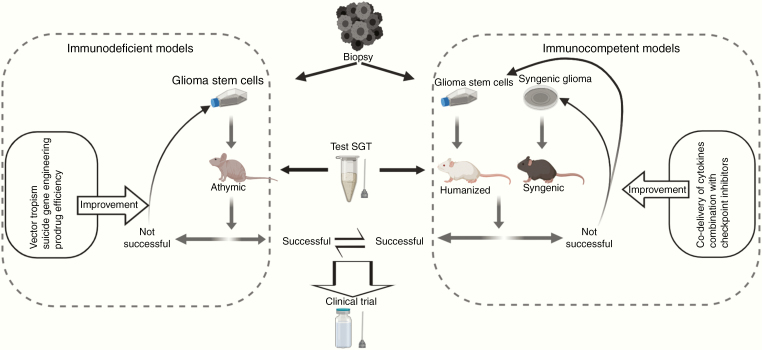
Outline of translational strategies to improve future SGTs. The choice of preclinical model systems is an important aspect for developing SGTs toward HGG treatment. GSCs are known to be the most clinically relevant preclinical models that recapitulate patient tumors very closely and thus a particular SGT should be tested in GSCs. If unsuccessful, the therapy should be subject to further improvements in different aspects as indicated. If successful, the therapy should be tested in immunocompetent models, for example, syngeneic glioma models and humanized models to analyze immune responses. If durable antitumor immune responses are detected, the particular SGT should be considered for clinical translation. Otherwise, additional treatments as combination with SGT can be considered to boost the immunostimulatory effect of a particular SGT. Combination with checkpoint inhibitors and co-delivery of immunostimulatory cytokines are important examples.

The immunosuppressive glioma microenvironment represents another clinical challenge. Although there are indications that the SGT-induced cell death is immunogenic, this has not been tested in detail, especially not in gliomas. Here, a lot can be learned from oncolytic viruses where an antitumor immune response is promoted by the vectors’ ability to induce ICD through oncolysis and thereby recruit an efficient antitumor IR.^92,93^ ICD might thus be a compulsory prerequisite for the engagement of an effective antitumor IR. More fundamental research is needed to identify the intrinsic nature of cell death, namely, immunogenic or immunosuppressive by the SGT systems in GSCs. If one SGT system fails to elicit strong ICD in GSCs (or in primary glioma cells), novel strategies or combinations could be designed to reroute the cell death mechanism. Unfortunately, the syngeneic animal models for glioma available today remain a serious drawback in this regard, because the immune microenvironment in these models differs substantially in composition from the one observed in HGG patients. The use of GSCs in humanized rodent models in this context may create valuable new information. Thus, a number of different preclinical models should be adopted to further develop SGT in the future and to achieve a better clinical translation procedure (Figure 4).

Combinatorial approaches using SGT with co-expression of immune-stimulating cytokines have been performed; however, these approaches might be too unspecific and are mostly directed toward T cells. Yet, T cell infiltration is scarce in glioblastoma, where immunosuppressive microglia and macrophages predominate. To improve future SGT approaches, the changes in the immune microenvironment under SGT should be analyzed more thoroughly. Based on this knowledge, specific shortcomings in the antitumor immune response during SGT could be detected and more targeted approaches could be developed.
